# Longitudinal Stretching for Maturation of Vascular Tissues Using Magnetic Forces

**DOI:** 10.3390/bioengineering3040029

**Published:** 2016-11-16

**Authors:** Timothy R. Olsen, Megan Casco, Austin Herbst, Grace Evans, Taylor Rothermel, Lauren Pruett, Jared Reid, Kelly Barry, Michael P. Jaeggli, Dan T. Simionescu, Richard P. Visconti, Frank Alexis

**Affiliations:** 1 Department of Bioengineering, Clemson University, 301 Rhodes Research Center, Clemson, SC 29634, USA; trolsen@clemson.edu (T.R.O.); mcasco@clemson.edu (M.C.); aherbst@clemson.edu (A.H.); gracee@clemson.edu (G.E.); trother@clemson.edu (T.R.); lpruett@clemson.edu (L.P.); jjreid@clemson.edu (J.R.); krbarry@clemson.edu (K.B.); mjaeggl@clemson.edu (M.P.J.); dsimion@clemson.edu (D.T.S.); 2 Department of Regenerative Medicine and Cell Biology, Medical University of South Carolina, 173 Ashley Avenue, 601 Basic Science Building, Charleston, SC 29425, USA; visconrp@musc.edu; 3 Department of Bioengineering, Institute of Biological Interfaces of Engineering, Clemson University, 401-2 Rhodes Engineering Research Center, Clemson, SC 29634, USA

**Keywords:** tissue engineering, spheroids, tissue fusion, magnetic nanoparticles, magnetic forces, tissue maturation

## Abstract

Cellular spheroids were studied to determine their use as “bioinks” in the biofabrication of tissue engineered constructs. Specifically, magnetic forces were used to mediate the cyclic longitudinal stretching of tissues composed of Janus magnetic cellular spheroids (JMCSs), as part of a post-processing method for enhancing the deposition and mechanical properties of an extracellular matrix (ECM). The purpose was to accelerate the conventional tissue maturation process via novel post-processing techniques that accelerate the functional, structural, and mechanical mimicking of native tissues. The results of a forty-day study of JMCSs indicated an expression of collagen I, collagen IV, elastin, and fibronectin, which are important vascular ECM proteins. Most notably, the subsequent exposure of fused tissue sheets composed of JMCSs to magnetic forces did not hinder the production of these key proteins. Quantitative results demonstrate that cyclic longitudinal stretching of the tissue sheets mediated by these magnetic forces increased the Young’s modulus and induced collagen fiber alignment over a seven day period, when compared to statically conditioned controls. Specifically, the elastin and collagen content of these dynamically-conditioned sheets were 35- and three-fold greater, respectively, at seven days compared to the statically-conditioned controls at three days. These findings indicate the potential of using magnetic forces in tissue maturation, specifically through the cyclic longitudinal stretching of tissues.

## 1. Introduction

The use of cellular spheroids in any bioprocessing process involves pre-processing, processing and post-processing. The first step in this three-step process is known as pre-processing, i.e., the preparation of the spheroids for use as the building blocks for tissue fabrication [1]. The second step, known as processing, entails the patterning of the cellular spheroids into the desired orientation and fusion of adjacent spheroids [1,2,3,4,5,6]. Finally, the tertiary, or post-processing step involves tissue maturation over a certain time via mechanical stimulation and the use of chemical maturagens [7,8,9,10]. Although the goal of tissue maturation involves improving the mechanical properties, ECM content and functionality of the developing tissues, conventional cell culturing, and post-processing methods can take months, or even years, to create such tissues with specific mechanical properties [11,12]. Consequently, there is a critical need to elucidate and accelerate tissue fusion and maturation for the purposes of producing tissue-engineered constructs on demand.

Methods to accelerate and enhance the fusion and maturation of vascular tissue scaffolds have involved the use of post-processing mechanical conditioning.

Specifically, using a 10% cyclic radical strain, at a 1 Hz frequency over a 24 h period, in individual studies Seliktar et al. and Schutte et al. enhanced the strength of tissue-engineered vascular mediums by 22%–57% and increased collagen content by 22%, when compared to statically-cultured controls [9,13,14]. In both studies, however, the parameters for optimal maturation (frequency, magnitude, time of stimulation) were neither defined nor standardized, nor was the longitudinal stretch placed upon the blood vessels analyzed [9,12,13,15]. It is known that almost all arteries in vivo experience longitudinal (axial) strain at magnitudes ranging from 40% to 65% [16,17,18]. In their study using tissue sheets composed of human dermal fibroblasts exposed to a dynamic longitudinal strain (10% strain, 1 Hz, 24 h per day) Gauvin and coworkers observed an increased expression of type I collagen and elastin when compared to static controls [7]. They also noted a 1.85-fold increase in the axial ultimate tensile strength and a 1.72-fold increase in the axial modulus, respectively, when compared to statically-cultured tissues. All of these studies suggest the importance of incorporating longitudinal strain into any future techniques for post-processing vascular tissues.

Initial studies have also been undertaken to test the concept of using magnetic forces to initiate longitudinal stretching for tissue maturation. Although magnetic nanoparticles (MNPs) have been used in cellular spheroids to align and pattern tissues, which are mediated by magnetic forces [1,5,19,20,21,22,23,24,25], the use of magnetic force for accelerating the maturation of vascular constructs has not been studied. Magnetic forces have been used successful, however, in the maturation of bone constructs within a period of 1–3 weeks, demonstrating the possibility of induced desired differentiation and an increased ECM production [26,27]. The rationale is that the application of these magnetic forces induces a mechanical stimulation of the tissues with MNP, thus resulting in a preferential cell alignment and deposition of an ECM. Magnetic force technologies are particularly effective in tissue engineering applications because they allow for the use of magnetic fields to precisely control and manipulate the tissues at a distance [1,5,22,23,24,28,29,30]. Although current patterning methods utilize molds, complex bioprinting systems, or layers of hydrogel to anchor spheroids, only passive contact between the spheroids is possible. However, the use of spheroids in magnetic force-based tissue engineering is an attractive option because the increased inter-spheroidal contact via magnetic forces can greatly accelerate their fusion. [1,5,22,23,24,29].

This paper describes of use of Janus magnetic cellular spheroids (JMCSs) in the mechanical maturation of tissues for vascular tissue applications. Through the Janus method of spheroid fabrication, the viability, functionality, and phenotypic characteristics of a cell remain stable over time, even though the cells and extracellular MNPs remain separate [4,5,6,22,23,24]. The objective of this work was to test the effects of magnetic forces on tissues composed of JMCSs with respect to the ECM deposition and mechanical properties over time. The hypothesis driving this research was that magnetic forces can mechanically stimulate tissues composed of JMCSs to stretch the tissues in a controlled, cyclic, uniaxial longitudinal direction for such evolutionarily-induced maturation. Understanding how these magnetic forces facilitate the maturation of tissues composed of JMCSs over time can provide the method by which tissues may be fabricated for specific applications.

## 2. Experimental Section

### 2.1. Cell Culture

Primary rat aortic smooth muscle cells (SMCs) and passage numbers under ten were used in all experiments. Dulbeco Modified Eagle Medium:F-12 (ATCC, 1:1, DMEM:F-12) supplemented with a 10% fetal bovine serum (Atlanta Biologicals, Flowery Branch, GA, USA) and a 1% penicillin-streptomycin-amphotericin (MediaTech, Inc., Manassas, VA, USA) was used to culture SMCs in monolayers at 37 °C and 5% of CO_2_ prior to spheroid assembly.

### 2.2. Spheroid Formation

Spheroids were prepared using a method previously developed in our lab [22,23,24]. Uniform volumes of solutions comprised of suspended iron oxide MNPs (Fe_3_O_4_, 20–30 nm, SkySpring Nanomaterials, Inc., Houston, TX, USA), collagen (Bovine, Type I, Life Technologies, Carlsbad, CA, USA), and cells in media were mixed and dispersed onto the inside tops of Petri dishes using our specific hanging drop technique into 15 μL droplets. Since the iron oxide magnetic nanoaparticles are heavier than the cells and ECM, gravity concentrates the particles and creates two separate domains within the spheroid, one domain with cells and ECM, and one domain with extracellular iron oxide nanoparticles. Spheroids were fabricated with 20,000 cells each and left to form for three days prior to use. Collagen was prepared according to the recommendations of the manufacturer and kept on ice before use. For long term studies, spheroids were placed into 96 non-tissue culture-treated well plates in the presence of cell culture media, with and without ascorbic acid supplementation (50 μg/mL).

### 2.3. Tissue Sheet Fabrication

A strip of magnets (2.5 mm outer diameter, 5 mm length, K and J Magnetics, Inc., Pipersville, PA, USA) were used to assemble our monolayer sheets composed of JMCSs. The dimensions of the tissue construct reflected those of the magnetic template, and were rounded at the ends of the construct. The resulting sheets were typically 2–3 spheroids thick, with a total tissue thickness ranging between 1 and 2 mm. Five magnets were attached to the underside of a six-well plate (Greiner Bio-One, Monroe, NC, USA) and a glass cover slip (micro cover glass 22 × 30 mm, VWR, Radnor, PA, USA) was positioned directly over the magnets within the plate to prevent any spheroid adherence. The wells were filled with cell culture media, and a pipette tip was then used to carefully align 1000 JMCSs of the same formulation on the coverslip. The product was then incubated at 37 °C and 5% CO_2_ for three days so that fusion could occur.

### 2.4. Histological Examination

Spheroids and tissue sheets were collected, fixed, and processed at different time points for histological examination [4,5]. Tissues were embedded into paraffin and samples were cut into 5 μm sections, followed by staining with either hematoxylin and eosin (H and E), or Masson’s trichrome stain.

### 2.5. Viability Analysis

After seven days of culture in both the presence and absence of dynamic conditioning, a LIVE/DEAD viability assay, performed according to the manufacturer’s protocol (Life Technologies), was used to collect and analyze tissue sheets. An EVOS fluorescent digital inverted microscope was then used to image the samples.

### 2.6. Immunohistochemistry

Four ECM markers were analyzed: collagen I, collagen IV, fibronectin and elastin. The following antibodies were used to stain for these markers: anti-collagen I antibody (1:200; Abcam; ab34710), anti-collagen IV antibody (1:200; Abcam; ab6586), anti-fibronectin (1:200; BD Biosciences; 610077), and anti-elastin (1:50; Abcam; ab23748). A BOND-MAX automated IHC machine (Leica) was used to analyze the prepared slides, and an automated Novocastra Bond Polymer Refine Detection system (Leica; DS9800) was used to stain and detect protein markers in the tissue samples. A Nikon AZ100 multizoom microscope was used to image the stained samples, and the results were compared to respective control samples that were only counterstained for nuclei.

### 2.7. Maturation with Magnetic Forces

After ten days of fusion, the magnetic templates underneath the well plates containing the fused magnetic cellular sheets were removed. The wells containing the fused magnetic cellular sheets were placed onto a platform inside an incubator set at 37 °C and 5% CO_2_. A rod magnet (30 mm) composed of stacked cylinder magnets (2.5 mm diameter, 5 mm length, pull force = 1.8 lbs, SuperMagnet Man) was placed directly underneath each magnetic cellular sheet on the platform. The platform containing the rod magnets was fixed to a linear actuator (L16-P Linear Actuator, Firgelli, Victoria, BC, Canada) that was controlled using a custom program built in LABVIEW (National Instruments). This program allowed for the controlled cyclic longitudinal translation of the rod magnets, which were used in the longitudinal cyclic stretching (i.e., the frequency, magnitude, and time of stretch) of the magnetic cellular sheets. The frequency and magnitude were 1 Hz and 10% respectively, and based upon previous studies involved with blood vessel tissue engineering [7,9,13,14].

### 2.8. Mechanical Testing with Atomic Force Microscopy

Atomic force microscopy (AFM) (Asylum MFP-3D atop an Olympus IX81 Spinning Disc Confocal system) was used to determine the Young’s modulus of statically- and dynamically-conditioned tissue sheets at three and seven days, respectively A cantilever (~0.12 N/m spring constant) with a borosilicate spherical tip (5 µm diameter) was used to indent the tissue sheet samples. The spring constant was calibrated in air. The cantilever deflection was measured and converted to a force measurement using the respective Hook’s spring constant of the tip [31,32]. In order to identify and maintain the contact point between the tip and tissue sample, the z-piezo displacement was constantly monitored and samples were visually inspected with scanning confocal microscopy [32]. The Hertz model and the slope of the force-displacement curves generated from MATLAB were used to calculate the modulus based on previous methods [32,33,34]. The extended curve was used due to the absence of such interactions, like adhesion, that make a determination of contact point impossible. Three indentation “sections” were used to analyze consistency throughout all of the tissue samples analyzed, indenting on the left, middle, and right sides of the tissues. At each of these locations, a minimum of five curves were used, making a minimum total of 15 curves per tissue tested [31]. A total of three tissue samples were tested for each group.

### 2.9. Western Blot for Collagen and Elastin Protein Quantification

Sheets of tissue, both stretched and non-stretched via magnetic forces, were collected after three and seven days, and flash frozen for storage. The samples were then immersed in a lysis buffer and sonicated to extract the protein, the content of which was quantified using spectroscopy. Each sample was then loaded into gels with a 10 μg/well. Regarding the primary antibodies, a rabbit polyclonal antibody against collagen type I was used to detect the level of collagen I content (Abcam, ab34710), a mouse monoclonal antibody against Hsp47 was used to detect the level of Hsp47 content (Calbiochem, M16.10A1) and a mouse monoclonal antibody against elastin was used to detect the level of elastin content (Santa Cruz Biotechnology, sc-374638). Chemiluminescence via peroxidase-conjugated horseradish secondary antibodies with Amersham’s ECL Prime Western Blotting Detection Reagent were used to detect protein band intensities, which were then quantified with ImageJ.

### 2.10. Collagen Quantification

A well-established assay, developed for quantitatively determining hydroxyproline was used here to determine the collagen content in the media that was produced by the tissue sheets [35]. The media was first collected, frozen, and then lyophilized, and samples were then hydrolyzed in 4 N sodium hydroxide at 120 °C for 2 h. Samples were neutralized with 1.4 N of citric acid and pH balanced to within a range of 7.2–7.6. A hydroxyproline standard curve was prepared for the calibration of unknown samples. An aliquot of 200 μL was taken from each sample for incubation with a Chloramine T solution for 15 min and then a *p*-dimethylaminobenzaldehyde solution for 15 min at 65 °C. Experimental samples were prepared in triplicate for the optical number readings at 550 nm. The samples on days three and seven were normalized to the collagen content of each respective group on Day 1.

### 2.11. Collagen Fiber Alignment

The collagen fiber alignment was characterized by measuring the angle of 50 collagen fibers from day seven dynamically-conditioned tissue sheet sections (*n* = 3), with respect to the axis of the applied magnetic cyclic longitudinal forces. A modified Masson’s trichrome stain was used for staining only the collagen fibers, and the sections were imaged using a Nikon AZ100 microscope. ImageJ was then used to measure the fiber angles, with respect to the origin of the direction of the magnetic forces.

### 2.12. Statistical Analysis

An analysis of variance (ANOVA) was next used to determine the presence of statistical differences amongst the treatment groups. A post hoc two tailed *t*-test was next used on any differences derived from the ANOVA to determine any significant differences between the analyzed treatments. The error bars on the graphs represent the standard deviation from the mean.

## 3. Results

**Figure 1:**
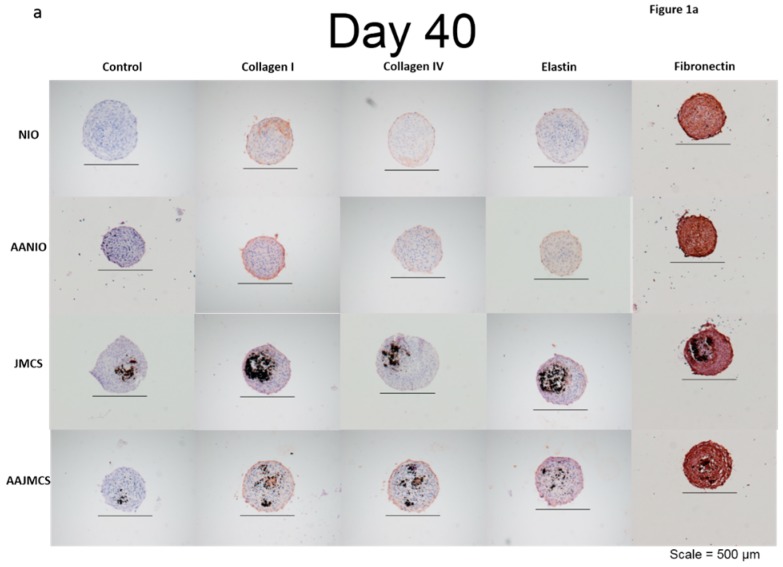
Immunohistochemistry staining of spheroids over time. Spheroids containing iron oxide MNPs and spheroids without MNPs were immunohistochemically examined for collagen I, collagen IV, elastin, and fibronectin after a 40-day incubation period in cell culture media. Spheroids with and without MNPs were also incubated in media supplemented with ascorbic acid, which is a known stimulant of collagen production. Each spheroid type demonstrated positive stains for each ECM marker when compared to their respective controls. A preferential cellular organization, as illustrated in the spherical geometry of the spheroids was observed (black = iron oxide, brown = positive stain).

Spheroids were incubated for 40 days in cell culture media, both with and without MNPs, and also with and without ascorbic acid supplementation (50 μg/mL). Ascorbic acid, which is a known evolutionary stimulant of collagen production, was used as the control for our spheroids both with and without MNPs. Results demonstrated that each spheroid type was immunopositive for collagen I, collagen IV, elastin, and fibronectin ECM proteins when compared to control samples only stained with hematoxylin (Figure 1a). After 40 days, the spheroids were organized, as depicted by their well-defined spherical shape and cellular organization (Figure 1a). The black spots in the images represent the iron oxide MNPs, and the brown color represents the positive immunostaining. The immunopositive area observed in JMCSs spheroids at day 40 were comparable to the “no iron oxide” (NIO) group supplemented with ascorbic acid, which is known to stimulate ECM production [11,36,37]. Additionally, results show that sections of JMCSs supplemented with ascorbic acid exhibited a higher collagen content at the day 40 time point, suggesting a synergistic effect between the iron oxide MNPs and the ascorbic acid for producing an ECM, as previously shown [6].

**Figure 2:**
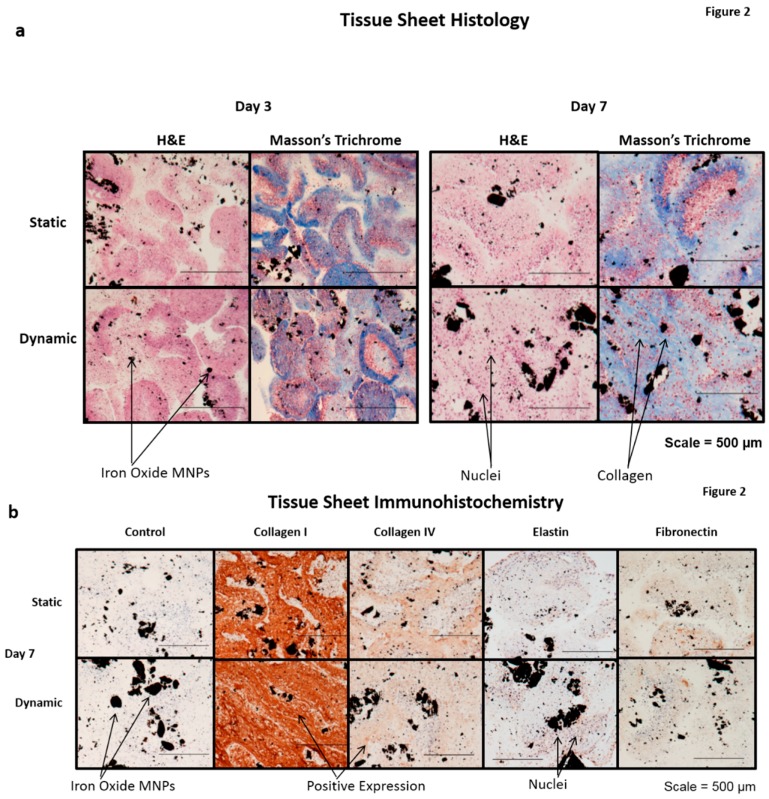
Tissue sheet histology. (**a**) Fused tissue sheets composed of JMCSs were either statically maintained on a magnetic template or dynamically conditioned using magnetic forces. After three and seven day time points, the sheets were collected, fixed, and processed for histological examination, with the representative H and E and Masson’s trichrome stains shown here: (black = iron oxide, blue = collagen, purple = nuclei); and (**b**) a comparison of the static and dynamic tissue sheets with the control tissue samples: all are immunopositive for collagen I, collagen IV, elastin, and fibronectin (black = iron oxide, brown = positive stain, purple = nuclei).

Magnetic forces were next used to pattern JMCSs into tissue sheets and to promote their fusion over time through the promotion of active contact between adjacent spheroids (Supplemental Figure S1a). After 10 days of fusion, tissue sheets (Supplemental Figure S1b) were either statically maintained on a magnetic template and dynamically conditioned using a cyclic longitudinal stretch mediated by magnetic forces for three or seven days (Supplemental Figure S1b). A live/dead stain procedure was performed on statically- and dynamically-cultured tissue sheets conditioned after seven days, thus demonstrating the presence of a large population of viable cells (Supplemental Figure S2), similar to previous live/dead staining of tissue sheets after three days of fusion [4]. A subsequent H and E histological examination also demonstrated the presence of cell nuclei throughout the statically- and dynamically-conditioned sheets at the three and seven day time points (Figure 2). A Masson’s trichrome stain also demonstrated the presence of a collagenous ECM throughout the statically- and dynamically-conditioned groups at the same three and seven day time points (Figure 2a). Results clearly demonstrated the presence of aligned collagenous fibers along the stretching direction in the tissue sheets that were dynamically conditioned for seven days, thus indicating that the cyclic longitudinal conditioning mediated by magnetic forces caused a remodeling of the ECM in response to the dynamic forces applied. The results of immunohistochemical staining also demonstrated that tissue sheets produced ECM over time, in that these statically- and dynamically-conditioned sheets were immunopositive for collagen I, collagen IV, elastin, and fibronectin at both the day three and day secen time points (Figure 2b).

**Figure 3:**
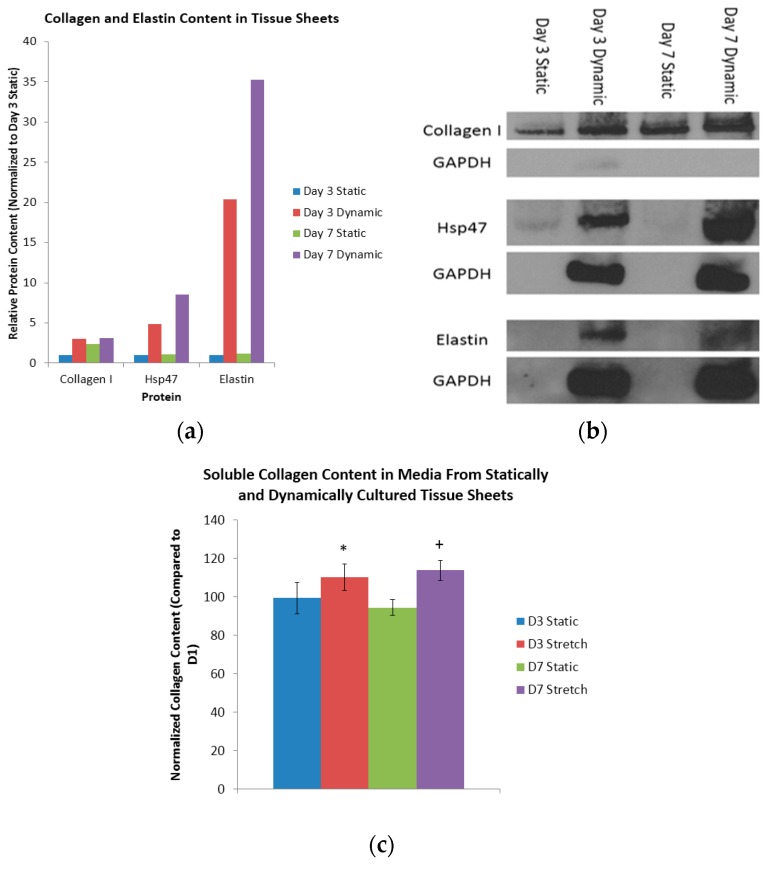
Insoluble and soluble protein content in tissue sheets. (**a**) Quantification of collagen I, Hsp47, and elastin content in tissue sheets with and without cyclic longitudinal stretching mediated by magnetic forces using Western blot; the results are normalized to day three static tissue sheets; (**b**) representative protein and glyceraldehyde 3-phosphate dehydrogenase (GAPDH) bands from the Western blots; and (**c**) the normalized soluble collagen content in the media of statically- and dynamically-conditioned tissue sheets after three and seven days: *n* = 3 (“*” and “+” indicate a statistical significance for the D3 and D7 stretch samples when compared to the D3 static control sample).

Dynamic mechanical conditioning was next used to induce collagen and elastin production in 2D cultures and 3D tissue constructs, with the 2D cultures composed of monolayers of cells adhered to either flat substrates or tissue culture plastic, and the 3D cultures composed of scaffolds, sheets, spheroids, and hydrogels [7,9,14,38]. A comparison of the collagen content of the day three static sheets with the (i) day seven static sheets; (ii) day three dynamic sheets; and (iii) day seven dynamic sheets indicated a respective 2.40-fold increase, a 3.03-fold increase, and a 3.09-fold increase in collagen (Figure 3a). Heat shock protein 47 (Hsp47), which is a collagen-binding glycoprotein that is localized in the endoplasmic reticulum, is a known mediator of collagen maturation [39]. Again, a similar comparison of the Hsp47 content of the day three static sheets with the (i) day seven static sheets; (ii) day three dynamic sheets; and (iii) day seven dynamic sheets indicated a respective 1.10-fold increase, a 4.84-fold increase and an 8.50-increase in Hsp47 (Figure 3a). Finally, a comparison of the elastin content in the day three static sheets with that of the (i) day seven static sheets; (ii) day three dynamic sheets; and (iii) day seven dynamic sheets indicated a respective 1.15-fold increase, a 20.4-fold increase and a 35.3-fold increase in elastin (Figure 3a). The respective protein bands from the Western blots are shown in Figure 3b. These results indicate that the dynamic longitudinal stretching, mediated by magnetic forces, caused an evolutionary increase in the collagen production when compared to statically-cultured controls.

Here, the total content of the soluble collagen was quantified at days one, three, and seven in the media collected from the statically- and dynamically-cultured tissue sheets (Figure 3c). When normalized to day one, the statically-conditioned tissue sheets at day three had a collagen content of 99% and 94%, respectively. Again when normalized to day one, the dynamically-conditioned tissue sheets at day seen had a collagen content of 110% and 113%, respectively. Finally, when compared to the static controls at the day three and day seven time points, the dynamically-conditioned tissues had a significantly higher collagen content (*p* < 0.05). It is clear from these results that the dynamic conditioning mediated by magnetic forces does indeed induce the production and reorganization of collagen.

**Figure 4:**
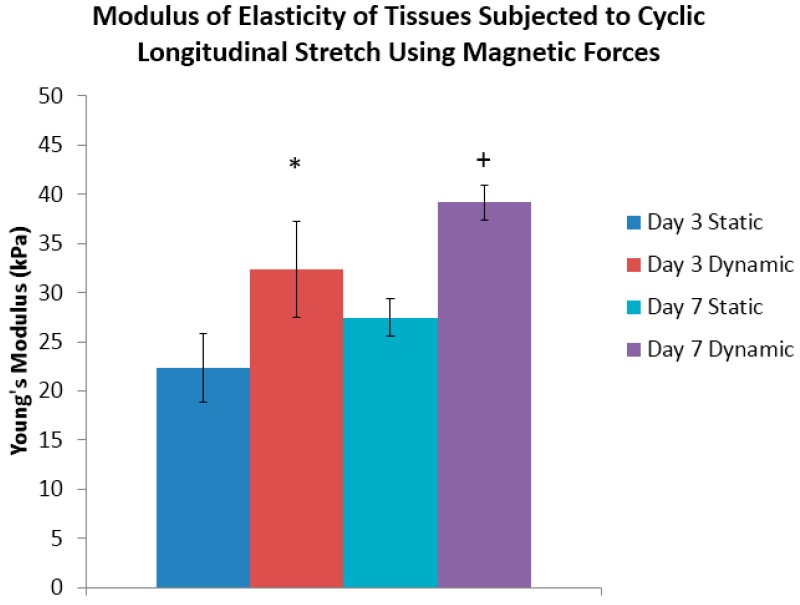
Tissue sheet mechanical properties. Atomic force microscopy of the tissue sheets demonstrating a significantly higher Young’s modulus of the dynamically-conditioned tissue sheets at both time points as compared to statically-conditioned controls: *n* = 3 tissue samples tested per group (“*” and “+” indicate the statistical significance).

The Young’s modulus of tissue sheets that were subjected to this cyclic longitudinal stretching that was mediated by magnetic forces were then compared to the statically-maintained tissue sheets at the three and seven day time points. In this phase of the work, a conditioning program with 1 Hz frequency, and a 10% magnitude of stretching over a 24 h period was used based upon established procedures and the physiological demands of other studies in the field [7,9,13,14,40]. AFM was used to determine the modulus of elasticity of conditioned tissue sheets at the end of three or seven days. A representative force-indentation curve from a day three statically-conditioned tissue sheet is shown in Supplementary Figure S3, from which the slope of this curve determines the modulus of elasticity. At the end of three days of conditioning, it was determined that the day three dynamically-conditioned tissue sheets had a Young’s modulus of 32 kPa, which was significantly greater than its statically-conditioned counterpart of 22 kPa (Figure 4). The results of the tests of the day seven dynamically-conditioned tissue sheets had a Young’s modulus of 39 kPa, which was significantly greater than the statically-conditioned counterpart of 27 kPa (Figure 4).

**Figure 5:**
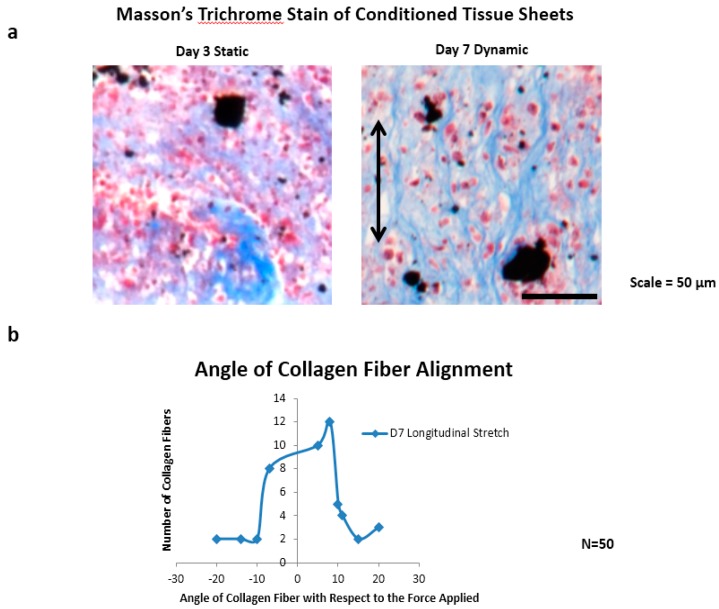
Collagen fiber alignment. (**a**) The presence of collagen fibers after seven days of cyclic longitudinal stretching via magnetic forces as compared to three days of static conditioning; and (**b**) the results of the measurement of the angle of 50 collagen fibers with respect to the applied magnetic forces: of the 50 fibers measured, 37 were within 10 degrees of the axis of the applied forces.

Collagen fiber alignment was observed in the direction of the applied magnetic cyclic longitudinal stretching forces for day seven samples only, using a modified Masson’s trichrome stain (Figure 5a). Three tissue sections were used to measure a total of 50 collagen fibers. Upon viewing the distribution of measured angles from ImageJ, there is a developing bell curve, with 30 of 50 angle measurements falling within eight degrees of the applied magnetic force, 37 of 50 angle measurements falling within 10 degrees of the applied magnetic force, and 13 of 50 falling outside of 10 degrees (Figure 5b).

## 4. Discussion

It is essential, when developing any tissue engineered vascular construct, to ensure that that the tissues contain collagen I, collagen IV, elastin, and fibronectin, all of which are important vascular ECM proteins [41]. Collagen I, which is present in all layers of blood vessels, is critical for stability of tissues [42]. Collagen IV, which is a major structural protein of basement membranes, will eventually form the structural skeleton of the developing tissue [42]. Elastin is distensible and provides elasticity to distribute stress throughout the vessel walls and collagen fibers. Similarly, fibronectin is essential for the development and facilitation of cell movement during the early migratory events in cell wall formation [42]. The continued expression of fibronectin over time during the maturation process in spheroids and sheets suggests its importance in maintaining tissue homeostasis [42]. Consequently, an increase in collagen IV and fibronectin expression in spheroids over 40 days would be expected to ensure the structural stability and organization with the evolution of the tissue [42]. Tissue sections of spheroids with iron oxide MNPs show a higher positive expression of collagen I when compared to controls without iron oxide, thus validating the hypothesis that iron oxide MNPs significantly enhance the production of collagen over time [6]. In previous work, we demonstrated that vascular smooth muscle cells maintain viability and cellular phenotype when formed into JMCSs [24]. Consequently, the development of viable and functional tissue engineered constructs means that vascular ECM markers must have long-term viability and must also have exhibit an immunopositive expression over a 40 day period.

Although an immunohistochemistry (IHC) failed to determine if the ECM deposition was more prevalent in our either statically- or dynamically-treated tissue samples, this process is limited in that the protein content can vary throughout; thus, a single 5 μm section may not be representative of the entire sample. The hydroxyproline and Western blot assays, however, are more accurate in determining a greater production of protein in the dynamically conditioned tissues compared to the static counterparts. Consequently, to increase the scope and accuracy of the quantitative protein content measurements of these two assays, entire tissue sheets were used rather than smaller sections. While ascorbic acid is known to enhance collagen production through ascorbate-dependent hydroxylation, its effect on elastin is not completely understood. In their use of 2D cultures of smooth muscle cells, de Clerck and coworkers determined that the deposition of insoluble elastin was inversely proportional to the concentration of ascorbic acid due to over-hydroxylation of tropoelastin, which is the precursor to insoluble elastin [37,43]. In their 2013 study of primary rat neonatal pulmonary fibroblasts Derricks and coworkers observed a dramatic increase in the production of elastin in response to ascorbic acid in 3D cell-gelfoam constructs [44]. Similarly, through our IHC and Western blot assays, we observed the production of elastin over time in individual cell spheroids and tissue sheets composed of cellular spheroids, and that our use of magnetic force-based longitudinal stretching did, indeed, enhance the elastin synthesis. As such, quantifying this enhanced synthesis will be the subject of future experiments under varying conditions.

The mechanical properties of native arteries and veins, which are well-established, have been used as the basis for developing and maturing tissues that mimic those mechanical properties [9,12,14,45]. Specifically, the use of spheroids and tissues composed of spheroids that characterize scaffold-free fabrication methods are expected to have a reduced mechanical property because of the need to endogenously produce an ECM. In their study of tissue sheets composed of human dermal fibroblasts exposed to static and dynamic longitudinal strain, Gauvin and coworkers observed, via Western blot analysis, an increased expression of type I collagen and elastin, as compared to their static controls [7]. They also noted a 1.856-fold increase in the axial ultimate tensile strength and a 1.724-fold increase in the axial modulus, respectively, when compared to the statically-cultured tissues.

Our results indicate that the cyclic longitudinal stretching mediated by magnetic forces provided the mechanical cues necessary for stimulating remodeling and reorganization of both the cells and the ECM according to the loads applied. Further, the subsequent dynamic conditioning increased the production of collagen, Hsp47 and elastin over the seven day period. Specifically, our Western blot data indicated that the glyceraldehyde 3-phosphate dehydrogenase (GAPDH) content for statically-cultured tissues was lower than that of the dynamically-conditioned tissues. GAPDH is a housekeeping protein that serves a function in glycolysis, gene transcription, RNA transport, DNA replication, and apoptosis [46]. Our results suggest that when compared to statically-maintained tissue sheets, dynamic conditioning may also serve a function in maintaining the expression of GAPDH, the content of which is dependent on the number of viable cells present in the tissues. This association suggests that when compared to statically cultured tissues, the dynamically-conditioned tissues contain more viable cells after the day three and seven time points. The results of a live/dead stain performed on day seven tissue sheets that were dynamically and statically cultured indicated a large population of viable cells in each group. The presence of this large viable cell population clearly indicates that the lack of GAPDH protein expression in statically-maintained tissues was from cellular death.

The tissue sheets were placed under influence of magnetic forces, and left to fuse for ten days, a fusion that was subsequently followed by either static or dynamic conditioning. The lack of magnetic forces holding the tissues intact suggests the possibility of a migration of cells out of the statically-conditioned tissue construct, thus decreasing the number of total cells and lowering the GAPDH expression. According to Riehl and collaborators, longitudinal stretching modulates cellular organization which, in turn, affects the functionality of the tissue engineered constructs (i.e., exerting an influence upon the protein expression, the cellular viability and the ECM deposition [47]. We attribute the cyclic longitudinal stretching mediated by magnetic forces as the reason for maintaining the expression of GAPDH, and for the increased production of both collagen and elastin.

The use of magnetic forces is a powerful method for engineering tissues in that it permits controlling, from a distance, the magnitude of force, frequency, and duration of treatment. Unlike passive conventional methods (e.g., using tissue molds in which spheroids are in loose contact with one another) MNPs can align magnetic forces and pattern spheroids and maintain active contact between spheroids to promote the optimum cell-cell interactions between adjacent spheroids to accelerate the fusion process over time. Here, the authors used a unique method of dynamic conditioning mediated by magnetic forces to enhance protein production and induce collagen crosslinking based on the appearance of aligned fibers. The development of a cross-linked collagenous network is critical for the successful creation of a scaffold free tissue engineered construct, in that it ensures structural integrity and acts as a scaffold for the cellular organization [12,16,48,49].

Iron has been shown to affect collagen on the cellular level by up-regulating collagen gene expression and, also, at the extracellular level by promoting and stabilizing collagen crosslinking [50]. Additionally, the gradual release of the ions within the tissue sheets over time, which is caused by the degradation of the iron oxide MNPs, may possibly up-regulate the production of collagen and promote the crosslinking of collagen produced by the tissues [23].

The presence of iron oxide within the MNPs of these tissue engineered constructs, however, may hinder the translation of this concept into the clinical setting. In a previous study, however, we coated our cellular MNP spheroids with biodegradable polymers which, in turn, caused a degradation of nearly 36% of the iron oxide over a 21 day period [21].

Our results from this study clearly indicate the possibility of the alignment of the collagen fibers as a causative factor for an increased modulus of elasticity, which was observed from the dynamic longitudinal stretching. Weidenhamer and coworkers created a method for aligning collagen fibers in tissue sheets composed of neonatal dermal fibroblasts after subjecting the samples to a 5% strain at a rate of 0.5 Hz over a period of three weeks [51]. A subsequent analysis of the stretched tissue determined that the axial alignment of all actin and collagen fibers were within 15 degrees of the axis of the applied strain [51]. In a similar study, Gauvin and coworkers demonstrated the efficacy of mechanical stretching with their successful alignment of collagen and actin in stretched tissue sheets for the alignment and organization of an ECM [7].

In our previous work, we successfully wrapped these tissue sheets, composed of fused JMCSs, around a silicone mandrel to form a tissue tube during the processing procedure [24]. Our future studies will entail the continued evolution of our post-processing methods and elucidating the mechanism behind those processes so that we can refine our engineered constructs made of spheroids to that of native tissues in terms of mechanical properties and ECM content. Specifically, those future studies will entail the creation of mechanically-conditioned tissue tubes (via magnetic forces) to study both the burst pressure and the strength of suture retention of those tubes. The post-processing conditions will be varied in terms of the frequency of stimulation, and the magnitude and duration of the stretching to which these tubes will be subjected.

Generating functional tissue engineered constructs is an absolute necessity for developing post-processing methods for enhancing the ECM deposition and the mechanical properties of tissues composed of cellular spheroids. In our 40 day experimental trials, we observed a continued expression, through JMCSs, of collagen I, collagen IV, elastin, and fibronectin, all of which are key vascular ECM proteins. Results also indicated that sheets of tissue composed of JMCSs will continue to produce these key proteins, indicating that this sheet tissue processing step enhances the scale of ECM production. The quantitative results demonstrated that the cyclic longitudinal stretching of the tissue sheets mediated by magnetic forces did indeed enhance the stiffness of the tissues over time. Additionally, the use of magnetic-force based engineering, in response to the forces applied, enhanced the production and alignment of collagen and elastin, thus making it a viable option for improving tissue bio-fabrication processes.

## Supplementary Materials

The supplementary materials can be accessed at http://www.mdpi.com/2306-5354/3/4/29/s1.
