# Supplementary material for: Longitudinal Stretching for Maturation of Vascular Tissues Using Magnetic Forces
Source: Bioengineering (Basel). 2016 Nov 16;3(4):29. doi: 10.3390/bioengineering3040029 (PMC5597272; doi:10.3390/bioengineering3040029)
Supplement: Supplementary file 1 [file bioengineering-03-00029-s001.pdf]

bioengineering

Supplementary Materials

Longitudinal Stretching for Maturation of Vascular Tissues Using Magnetic Forces

Timothy R. Olsen, Megan Casco, Austin Herbst, Grace Evans, Taylor Rothermel, Lauren Pruett, Jared Reid, Kelly Barry, Michael P. Jaeggli, Dan T. Simionescu, Richard P. Visconti and Frank Alexis

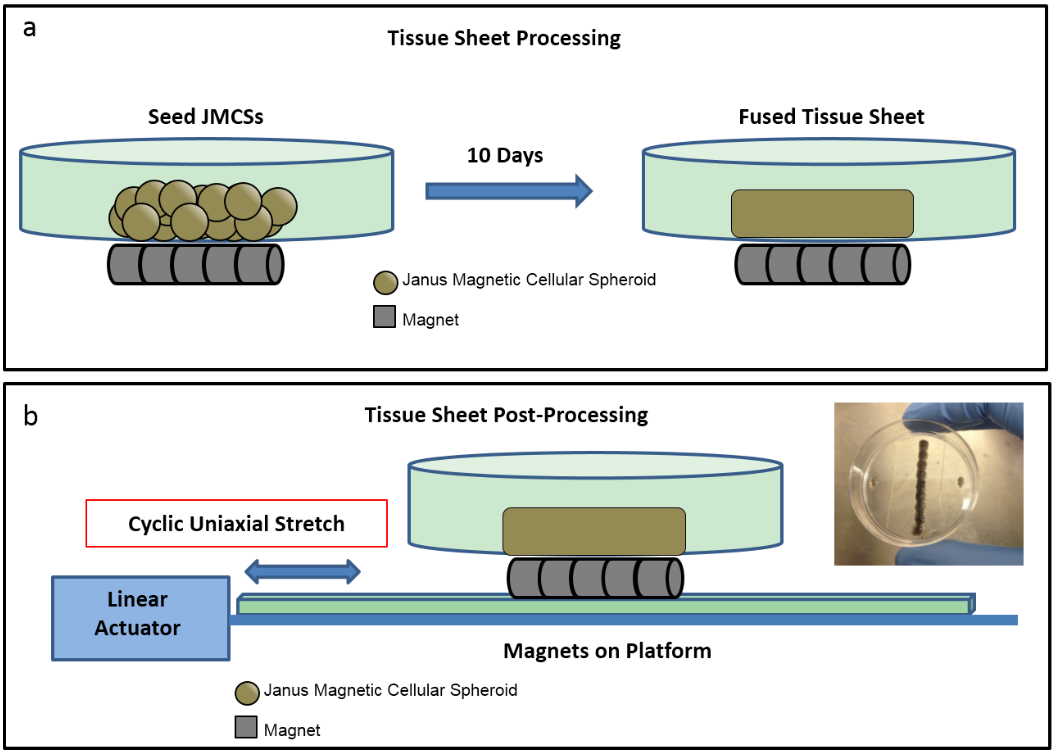

Figure S1. Processing and Post-Processing of Tissue Sheets Composed of JMCSs. (**a**) Schematic depicting tissue sheet processing. 1000 JMCSs were formed for three days using the hanging drop method. Spheroids were then seeded into a 6 well plate with a glass slide on the inside bottom surface. Magnets attached to the bottom of the 6 well plate allow for patterning of the spheroids into a rectangular sheet. Sheets were allowed to fuse for 10 days. (**b**) Schematic depicting tissue sheet post-processing. The magnetic templates were removed from the bottom of the well plates containing fused tissue sheets. The well plates containing the fused sheets were placed onto a platform inside an incubator. Using a linear actuator, an array of magnets was translated in a cyclic, uniaxial direction beneath the fused tissue sheets. The device allows for precise control over frequency, magnitude of stretch and duration of conditioning.

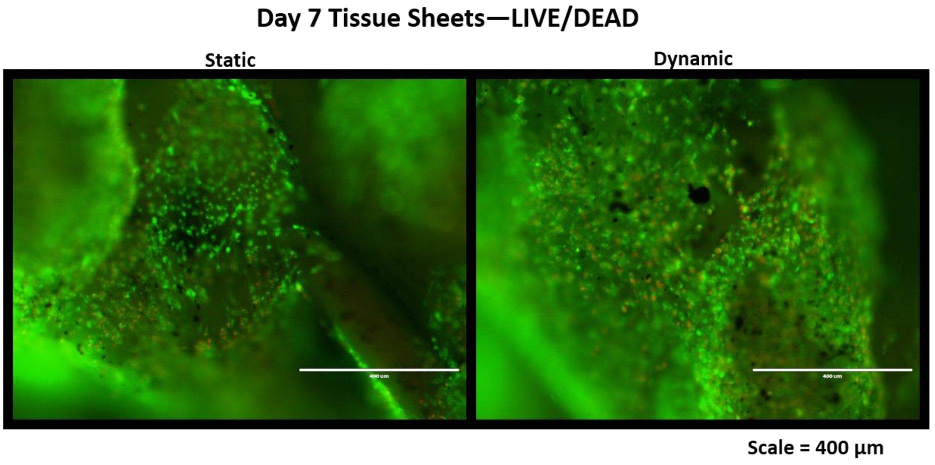

Figure S2. LIVE/DEAD Images of Tissue Sheets at Day 7. Images display LIVE/DEAD results of tissue sheets without (left) and with (right) cyclic longitudinal stretching mediated by magnetic forces after 7 days of culture. Scale = 400 µm.

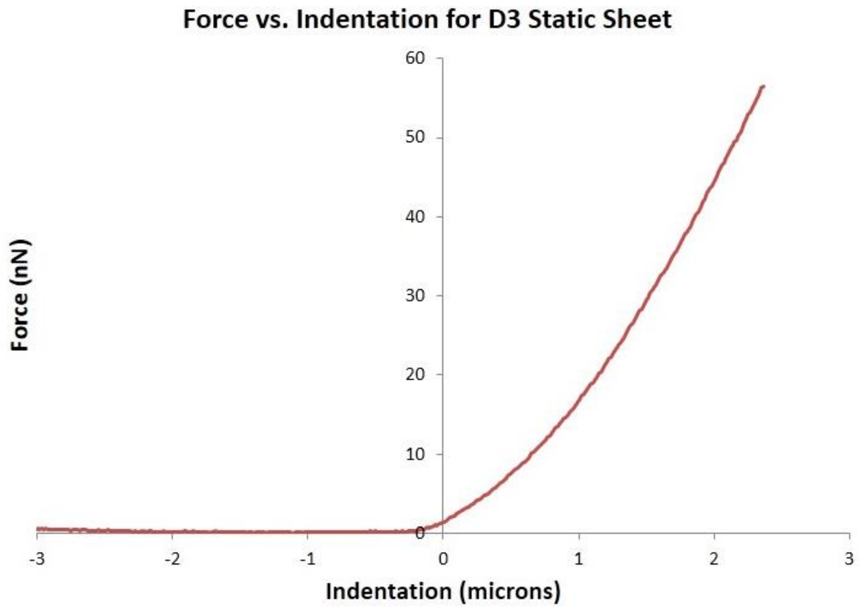

Figure S3. Force vs. Indentation Curve for D3 Static Tissue Sheet. Representative force-indentation curve, obtained from AFM, for a day 3 statically conditioned tissue sheet.

© 2016 by the authors. Submitted for possible open access publication under the terms and conditions of the Creative Commons Attribution (CC-BY) license (http://creativecommons.org/licenses/by/4.0/).
